# Electrospun Silk Fibroin Scaffolds for Tissue Regeneration: Chemical, Structural, and Toxicological Implications of the Formic Acid-Silk Fibroin Interaction

**DOI:** 10.3389/fbioe.2022.833157

**Published:** 2022-01-27

**Authors:** Marco Biagiotti, Giulia Alessandra Bassani, Anna Chiarini, Valentina Teodolinda Vincoli, Ilaria Dal Prà, Cesare Cosentino, Antonio Alessandrino, Paola Taddei, Giuliano Freddi

**Affiliations:** ^1^ Silk Biomaterials S.r.l, Lomazzo, Italy; ^2^ Department of Surgery, Dentistry, Pediatrics and Gynecology, Human Histology and Embryology Unit, Medical School, University of Verona, Verona, Italy; ^3^ NMR Center, Ronzoni Institute, Milano, Italy; ^4^ Department of Biomedical and Neuromotor Sciences, University of Bologna, Bologna, Italy

**Keywords:** silk fibroin, formic acid, spectroscopy analysis, toxicology, SILKBridge^®^ nerve conduit

## Abstract

The dissolution of *Bombyx mori* silk fibroin (SF) films in formic acid (FA) for the preparation of electrospinning dopes is widely exploited to produce electrospun SF scaffolds. The SILKBridge^®^ nerve conduit is an example of medical device having in its wall structure an electrospun component produced from an FA spinning dope. Though highly volatile, residual FA remains trapped into the bulk of the SF nanofibers. The purpose of this work is to investigate the type and strength of the interaction between FA and SF in electrospun mats, to quantify its amount and to evaluate its possible toxicological impact on human health. The presence of residual FA in SF mats was detected by FTIR and Raman spectroscopy (new carbonyl peak at about 1,725 cm^−1^) and by solid state NMR, which revealed a new carbonyl signal at about 164.3 ppm, attributed to FA by isotopic ^13^C substitution. Changes occurred also in the spectral ranges of hydroxylated amino acids (Ser and Thr), demonstrating that FA interacted with SF by forming formyl esters. The total amount of FA was determined by HS-GC/MS analysis and accounted for 247 ± 20 μmol/g. The greatest part was present as formyl ester, a small part (about 3%) as free FA. Approximately 17% of the 1,500 μmol/g of hydroxy amino acids (Ser and Thr) theoretically available were involved in the formation of formyl esters. Treatment with alkali (Na_2_CO_3_) succeeded to remove the greatest part of FA, but not all. Alkali-treated electrospun SF mats underwent morphological, physical, and mechanical changes. The average diameter of the fibers increased from about 440 nm to about 480 nm, the mat shrunk, became stiffer (the modulus increased from about 5.5 MPa to about 7 MPa), and lost elasticity (the strain decreased from about 1 mm/mm to about 0.8 mm/mm). Biocompatibility studies with human adult dermal fibroblasts did not show significant difference in cell proliferation (313 ± 18 and 309 ± 23 cells/mm^2^ for untreated and alkali-treated SF mat, respectively) and metabolic activity. An in-depth evaluation of the possible toxicological impact of residual FA was made using the SILKBridge^®^ nerve conduit as case study, following the provisions of the ISO 10993-1 standard. The Potential Patient Daily Intake, calculated from the total amount of FA determined by HS-GC/MS, was 2.4 mg/day and the Tolerable Exposure level was set to 35.4 mg/day. This allowed to obtain a value of the Margin of Safety of 15, indicating that the amount of FA left on SF mats after electrospinning does not raise concerns for human health.

## 1 Introduction

The use of *Bombyx mori* silk fibroin (SF) as a biomaterial of choice for the development of medical applications is a growing field of study (Holland et al., 2019). A variety of SF material formats has been proposed for manufacturing scaffolds and devices, spanning from native microfibers to films, hydrogels, sponges, nanofibers, micro- and nano-particles, etc. (Rockwood et al., 2011) and their combinations. A preliminary dissolution of native SF fibers is usually required to obtain regenerated scaffolding materials. The range of solvents available includes mineral and organic acids, organic solvents, ionic liquids, concentrated solutions of mineral salts, as well as different water/solvent/salt mixtures (Koeppel and Holland, 2017). In the 50s of the last century Earland and Raven (1954) proposed the use of formic acid (FA), mixed with small quantities of water and inorganic salts (e.g. CaCl_2_), for the dissolution of SF fibers until a concentration of 20% w/v. More recently, Zhang et al. (2014) reconsidered the use of the FA-CaCl_2_ solvent system to dissolve SF fibers without destroying their inherent nanofibrillar texture. The resulting solution was used to produce high strength and high-quality films and electrospun mats (Liu et al., 2015; Zhang et al., 2015a). FA-CaCl_2_ has also been used to spin regenerated microfibers by dry- (Yue et al., 2014) and wet-spinning (Zhang et al., 2015b). Authors claimed the obtainment of high-quality fibers with a hierarchical structure resembling the native one. The same solvent has been applied to the dissolution and regeneration of wild silk fibroins of the genus *Antheraea* and *Philosamia* (Xue et al., 2019). Changing the salt from CaCl_2_ to LiBr allowed producing electrospun SF mats with satisfactory mechanical stability, good biocompatibility, slow degradability, and promising new bone regeneration ability (Lu et al., 2015). Refinement of the FA-LiBr solvent system allowed controlling the degree of hydrogen bonding among the silk fibroin molecules composing the nanofibers so that they kept an amorphous state and remained stable in aqueous solution even after removal of FA and LiBr by dialysis (Dong et al., 2016).

Pure FA does not dissolve native SF fibers, while it is an effective solvent for regenerated SF materials like films (Um et al., 2001; Um et al., 2003). The route of dissolving SF films for the preparation of electrospinning dopes has been widely exploited to produce electrospun SF mats (Kim et al., 2003). Zhang et al. (2012) investigated the mechanism of nanofiber formation. They reported the effect of using electrospinning solutions with different nanostructures (nanospheres or nanofilaments) on the spinnability and diameter of electrospun fibers. FA is a highly volatile solvent, the most part of which evaporates during electrospinning. Residual FA remaining in the nanofibrous mat can be partly removed during the post-spinning washing/consolidation step, which is usually performed with water-alcohol solutions to achieve β-sheet crystallization and insolubilization of as-spun mats (Min et al., 2004). However, the elimination of the solvent is never complete, and a small amount remains trapped into the bulk of the nanofibers as shown by the presence of a characteristic FTIR carbonyl band (Um et al., 2001; Kim et al., 2003; Um et al., 2003; Wadbua et al., 2010; Zhang et al., 2012; Liu et al., 2015; Lu et al., 2015; Dong et al., 2016). We have recently developed an SF-based medical device, the SILKBridge^®^ nerve conduit, which is currently under clinical investigation (ClinicalTrials.gov identifier: NCT03673449). The conduit has a three-layered wall structure consisting of a braid made of native silk fibers as intermediate layer, sandwiched between two layers of electrospun nanofibers (Alessandrino et al., 2019). The latter are produced from an FA-based spinning dope. As expected, the FTIR spectra of the electrospun layers gave evidence of the presence of residual FA trapped into their matrix (Alessandrino et al., 2019).

The path taking a medical device from lab to clinic is marked by a series of regulatory obligations that impose several activities aimed at ensuring patient safety and health as primary asset that must not be jeopardized in any kind of approach aimed at solving clinical problems. Acquisition of raw materials in accordance with well-established quality assurance programs, rigorous control of the robustness of the manufacturing process, implementation and execution of on-bench and *in vitro* testing programs to ensure compliance of the device with the established specifications, thorough biocompatibility evaluation and execution of relevant *in vivo* functional tests using appropriate animal models are some of the necessary steps to allow a device accessing clinical trials. In this context, the evaluation of the potential toxicological risks associated with leachable substances released by a medical device in the surrounding tissues is an important step for the identification and quantification of the biological hazards related to its use. Therefore, the fate of processing aids used during manufacturing, such as FA, must be deeply investigated within a risk analysis framework which requires that not only their amount, but also the type and strength of the interaction with the polymeric matrix of the scaffold, and the conditions that may favor their release and diffusion into the surrounding tissues during implantation are elucidated.

To the best of our knowledge, the amount of FA and the nature and strength of its interaction with SF has never been investigated in detail. More important, the biological impact of residual FA has never been evaluated from a proper toxicological prospect as prescribed by in force regulations for medical devices (e.g. ISO 10993-1). Several studies have dealt with the *in vitro* (Min et al., 2004; Ki et al., 2007; Meechaisue et al., 2007; Ki et al., 2008; Marelli et al., 2009; Marelli et al., 2010; Park et al., 2010; Griffanti et al., 2019) and *in vivo* (Kim et al., 2005; Park et al., 2010; Cattaneo et al., 2013; Hu et al., 2013; Navone et al., 2014; Catto et al., 2015) biocompatibility of electrospun SF scaffolds produced from FA dopes. The good biocompatibility results reported so far support the promising biological performance of electrospun SF scaffolds. However, the clinical translation of these results cannot disregard an exact and thorough evaluation of the toxicological impact of leachable substances like FA that may be released at the implant site either because of diffusion phenomena and/or during the degradation of the scaffold itself. The purpose of this work is to investigate the nature of the interaction between SF and FA in electrospun mats. Different spectroscopic techniques (ATR-FTIR, Raman, ^13^C CP/MAS NMR) were used for the characterization of SF mats. The amount of FA was evaluated by chromatographic techniques (HS-GC/MS). The effect of an alkali treatment (Kishimoto et al., 2018) on the morphological, chemical, physical, and mechanical properties, as well as on the biocompatibility of electrospun mats was also investigated. The SILKBridge^®^ nerve conduit was used as case study to present a detailed evaluation of the possible toxicological impact of the release of FA during implantation in humans.

## 2 Materials and Methods

### 2.1 Materials Preparation

#### 2.1.1 Production of Electrospun SF Mats

Electrospun SF mats were produced by electrospinning using pupae-free silk cocoons as starting material. Cocoons were degummed in autoclave at 120°C for 20 min and extensively washed with water. Pure SF fibers were dissolved with a 9.3 M LiBr aqueous solution at 60°C for 3 h. The salt was removed by dialysis and aqueous SF was cast in Petri dishes at 35°C in a ventilated oven until complete evaporation of water. SF films thus obtained were dissolved in formic acid (FA) at 8% w/v concentration to prepare the electrospinning dope. ^13^C-enriched SF mats were electrospun from an FA solvent system containing 10% v/v^13^C-formic acid (Sigma-Aldrich, product # 279,404). Electrospinning was performed using the following experimental parameters: potential difference 25 kV, flow rate 0.8 ml/h, spinneret-collector distance 15 mm. As spun SF mats were consolidated by immersion in 80% v/v ethanol for 20 min at room temperature. Afterwards, they were washed overnight in distilled water at 37°C, under mild agitation, and dried at room temperature. For the alkali treatment, SF mats were immersed in aqueous Na_2_CO_3_ at different concentrations, 0.75, 1.5, and 3 M, at room temperature for different times, up to 16 h. Alkali-treated SF mats were then extensively washed in distilled water until neutrality and dried.

#### 2.1.2 Production of SILKBridge® Nerve Conduit

The SILKBridge^®^ nerve conduit was manufactured as previously reported (Alessandrino et al., 2019). Briefly, two electrospun layers were assembled onto the inner and outer faces of a tubular textile braid according to a patented process (Alessandrino, 2016). Coupling of the textile layer with the two electrospun layers was made by means of two different welding media: 1) a solution of ionic liquid (1-ethyl-3-methylimidazolium acetate; EMIMAc; #51053, Sigma-Aldrich) in water (EMIMAc/water 80/20% v/v); 2) a solution of 15% w/w SF in EMIMAc. After electrospinning, the hybrid tubular structure was consolidated by immersion in 80% v/v ethanol for 30 min at room temperature, followed by overnight washing with distilled water and drying. The device was further purified by microwave aided extraction with ethanol to remove processing aids, packaged under a laminar flow cabinet and sterilized with ethylene oxide (EtO).

### 2.2 Spectroscopic Characterization

#### 2.2.1 Attenuated Total Reflectance-Fourier Transform Infrared Spectroscopy (ATR-FTIR)

ATR-FTIR spectra were measured with an ALPHA FTIR spectrometer (Bruker) equipped with an ATR Platinum Diamond accessory, at a resolution of 4 cm^−1^, in the infrared range 4,000-400 cm^−1^. Spectra were corrected with a linear baseline and normalized to the CH_2_ bending peak at about 1,445 cm^−1^. This peak was selected because it is not sensitive to SF molecular conformation. Band intensity ratios were calculated as peak heights.

#### 2.2.2 Raman Spectroscopy

Raman spectra were recorded on a Bruker MultiRam FT-Raman spectrometer equipped with a cooled Ge-diode detector. The excitation source was a Nd^3+^-YAG laser (1,064 nm) in the backscattering (180°) configuration. The focused laser beam diameter was about 100 μm and the spectral resolution 4 cm^−1^. The reported spectra were recorded with a laser power at the sample of about 140 mW.

#### 2.2.3 ^13^C CP/MAS NMR Spectroscopy

^13^C CP/MAS NMR spectra were recorded on a Bruker Avance 300 spectrometer running at 75.47 MHz, using a 4 × 21 mm cylindrical zirconium rotor spun at 11,000 Hz. The ^13^C cross-polarization magic angle spinning (CPMAS) NMR spectra were acquired using recycle delay of 8 s, ^1^H 90 pulse length of 3.5 μs, 1 min contact time, acquisition time of 30 ms and from 1K to 4K scans. The chemical shifts were recorded about tetramethylsilane *via* benzene as a secondary reference.

### 2.3 Chemical Characterization

#### 2.3.1 Amino Acid Analysis

The amino acid composition was determined after acid hydrolysis with 6 N HCl at 105°C, under vacuum, for 24 h. Free amino acids were quantitatively determined by Ion Exchange Chromatography (Aminoanalyzer Biochrom Bio30+, Erreci, Milan, Italy), by elution with the lithium buffer system on a cation exchange resin column (High Pressure PEEK column packed with Ultropac eight cation exchange resin). Detection was made at 570 nm after post-column derivatization with ninhydrin. External standard calibration (Sigma, cod. AA-S-18) was used to quantify the amino acids. Samples were analyzed in duplicate.

#### 2.3.2 Quantitative Determination of FA

The quantitative determination of FA was performed by Head-Space Gas Chromatography/Mass Spectrometry (HS-GC/MS) (Bursova et al., 2015; Ghorbani et al., 2018). 10 mg of sample were finely chopped and transferred into a 10 ml glass vial for HS-GC. 4 μl of internal standard (419.6 mg/ml of acetic acid in water) and 200 μl of 6.7% v/v sulfuric acid in 1-propanol were added and the sample vial was incubated at 80°C for 30 min to derivatize formic and acetic acid to propyl formate and acetate, respectively. The HS-GC/MS analysis was performed with a GC-MS Shimadzu mod 2010 Plus equipped with a MS detector QP2010 Ultra, an Autosampler AOC 5000 Plus Shimadzu, and an Agilent HP-5ms Ultra Inert column (30 m × 0.25 mm; film thickness 0.25 μm). Other analytical conditions were as follows: carrier gas Helium at 1 ml/min; incubation temperature 80°C; incubation time 30 min; split less injector temperature 200 °C; temperature program: 40°C for 8.5 min—from 40°C to 260°C at 40°C/min—at 260°C for 2 min; injection volume 250 μl; transfer line temperature 280°C; source temperature 200°C; E = 70 eV; SIM acquisition mode. Target ions were propyl formate, m/z 42 and propyl acetate, m/z 61. Samples were analyzed in duplicate.

Free FA was analyzed as before, avoiding the derivatization step with sulfuric acid in 1-propanol and using an Agilent VF-MAXms column (30 m × 0.25 mm; film thickness 0.25 μm) for the separation of the compounds. The temperature program was: 40°C for 1 min—from 40°C to 230°C at 10°C/min—at 230°C for 1 min. Target ions were formic acid, m/z 46 and acetic acid, m/z 60. Samples were analyzed in duplicate.

### 2.4 Morphological Characterization

Morphological analyses were performed by Scanning Electron Microscopy (SEM, Zeiss EVO MA10) on Au/Pd sputter coated samples (Desk IV, Denton Vacuum, LLC), at 10 kV acceleration voltage, 100 μA beam current, and 15 mm working distance. For the determination of the diameter of the electrospun fibers, 10 fields at high magnification (×20,000) were randomly selected and the diameter of the fibers was measured using the image analysis tool of the SEM software.

### 2.5 Thermal Characterization

Thermal properties were determined by Differential Scanning Calorimetry (DSC) with a DSC 3500 Sirius (Netzsch). The sample (3–5 mg) was closed in an Aluminum pan and subjected to a heating cycle from 50°C to 400°C, at a heating rate of 10°C/min, under N_2_ atmosphere (flow rate: 20 ml/min).

### 2.6 Mechanical Characterization

Mechanical properties were measured in the tensile mode, using an All Electric Dynamic Test Instrument ElectroPuls E3000 (Instron), equipped with a load cell of 250 N and a thermostatic bath (BioPuls). SF mats were cut in strips of 10 × 30 mm, clamped on the machine at 10 mm gauge length, conditioned in the bath at 37 °C for 5 min, and tractioned in the submerged state at 10 mm/min crossbar rate. For each sample six specimens were measured and averaged.

### 2.7 *In Vitro* Biocompatibility

Sterile SF mats were placed inside 12-well plates (Falcon-Becton Dickinson) containing 0.4 ml of Dulbecco’s modified Eagle’s Minimum Essential Medium (DMEM; Sigma) and kept in place with stainless steel rings. Human Adult Dermal Fibroblasts (HADFs; ScienCell Research Laboratories, Carlsbad, CA, United States) were seeded on electrospun SF mats and polystyrene surfaces (control) and kept at 37°C in air 95% v/5% v CO_2_ for 3, 6, and 9 days. The growth medium used was 90% v DMEM, fortified with inactivated fetal bovine serum (FBS, 10% v; Thermo Fisher), and antibiotics. Every 3 days the growth medium was changed with fresh one and the cell-conditioned media collected and stored at −80°C to be later analyzed. Cell numbers were determined by the Quant-iT PicoGreen dsDNA Kit (Molecular Probes) at 3, 6 and 9 days of incubation. *d* -glucose uptake was assessed by means of a glucose oxidase assay using the Amplex^®^ Red Glucose/Glucose oxidase Assay Kit (Invitrogen). All the tests were performed in triplicate and repeated in three separate experiments. Data were expressed as mean values ± S.E. and their level of statistical significance assessed by one-way ANOVA followed by Holm-Sidak’s *post hoc* test. A *p* value <0.05 was taken as significant.

## 3 Results

### 3.1 Spectroscopic Characterization

**FIGURE 1:**
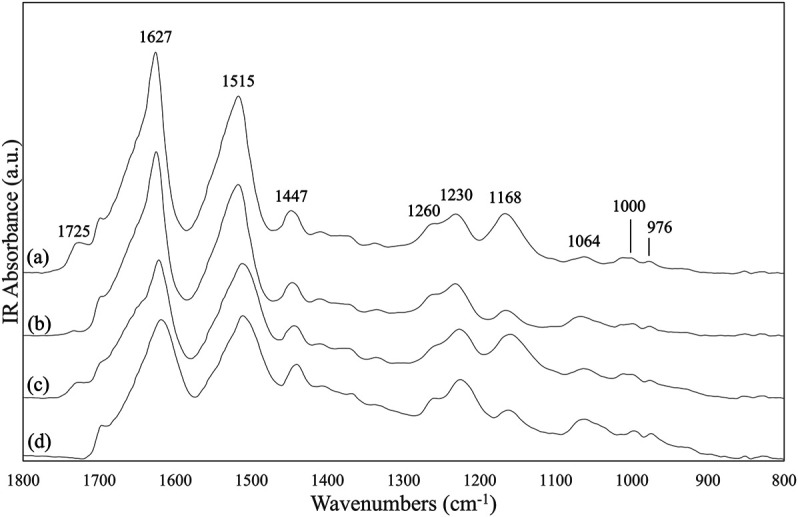
ATR-FTIR spectra in the 1,800-800 cm^−1^ range of: **(A)** electrospun SF mat; **(B)** SF mat treated overnight with 3 M Na_2_CO_3_; **(C)** SF mat after solubilization with LiBr, dialysis, and casting; **(D)** native SF fibers (reference).

The FTIR spectrum of the SF mat showed a band at 1,725 cm^−1^ attributable to the νC=O of FA (Figure 1A). As expected, this band was absent in the reference SF fibers (Figure 1D). In addition to the new carbonyl band, the skeletal stretching bands falling at 1,168 cm^−1^ and at 1,064 cm^−1^ (νC-O-C and νC-O vibrations, respectively) changed their intensity with respect to the reference material. The former band increased while the latter decreased. The behavior of the carbonyl and skeletal stretching bands was investigated as a function of different post-treatments. Dissolution with 9.3 M LiBr, followed by dialysis and casting, gave a reconstituted film with the FA carbonyl and skeletal bands still present with the same intensity of the original SF mat (Figure 1C), confirming the stability of the FA-SF bond. On the other hand, the overnight treatment with 3 M Na_2_CO_3_ (Figure 1B) (Kishimoto et al., 2018) significantly decreased the band at 1,725 cm^−1^, and those at 1,168 cm^−1^ and 1,064 cm^−1^ regained the shape and intensity characteristic of the reference material.

**FIGURE 2:**
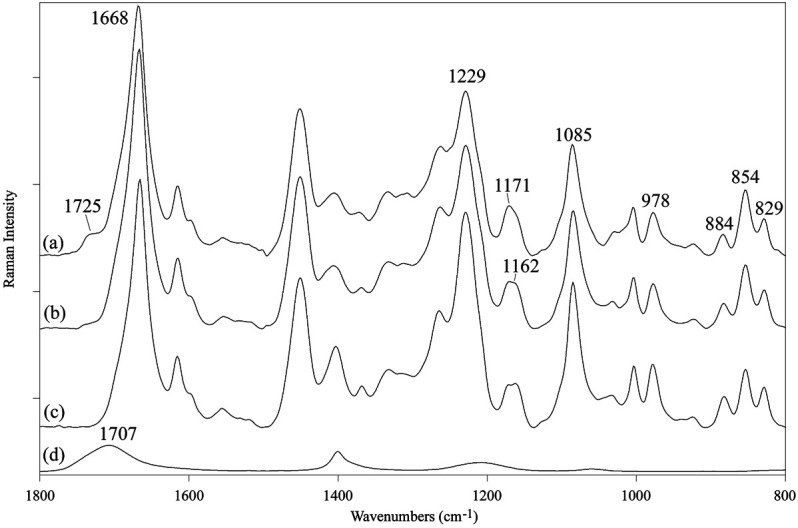
Raman spectra in the 1,800-800 cm^−1^ range of: **(A)** electrospun SF mat, **(B)** SF mat treated with 3 M Na_2_CO_3_, **(C)** native SF fibers (reference), **(D)** pure formic acid (reference).

The Raman spectrum of the SF mat showed the FA carbonyl band at 1,725 cm^−1^, although less intense with respect to FTIR (Dollish et al., 1973), and an increased spectral intensity at 1,171 cm^−1^ (Figure 2A), in good agreement with FTIR results. Upon treatment with aqueous Na_2_CO_3_ the band at 1,725 cm^−1^ decreased and only a weak feature was still detectable in the spectrum (Figure 2B). Accordingly, the band at 1,171 cm^−1^ detectably decreased in intensity and the overall spectral profile became very similar to that of the reference SF fibers (Figure 2C). Pure FA showed a prominent carbonyl band at 1,707 cm^−1^ (Figure 2D).

**FIGURE 3:**
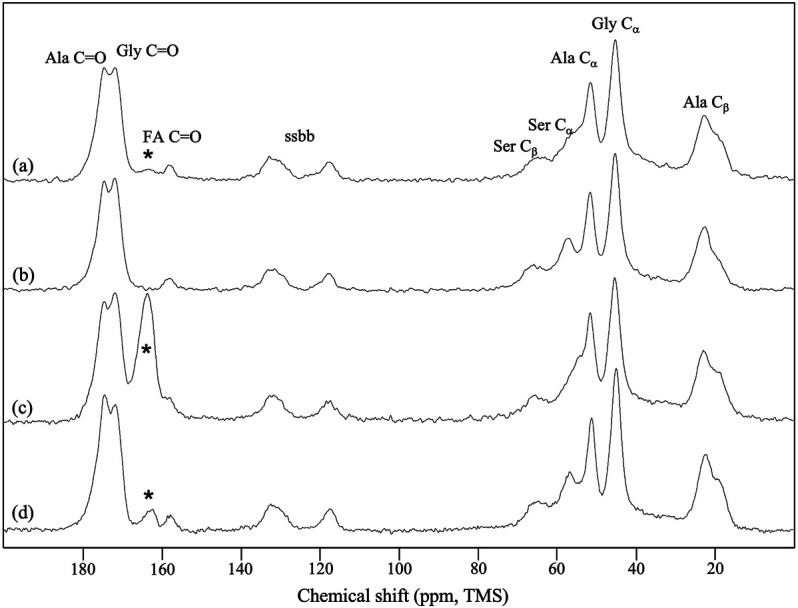
^13^C CP/MAS NMR spectra of: **(A)** electrospun SF mat; **(B)** native SF fibers (reference); **(C)** SF mat electrospun from a FA dope containing 10% v/v ^13^C-formic acid; **(D)** SF mat **(C)** treated overnight with 3 M Na_2_CO_3_. “ssbb”: side bands. The asterisks indicate the peak attributed to the C=O chemical shift of SF-bound formic acid.

The ^13^C CP/MAS NMR spectrum of the SF mat (Figure 3A) differed from the reference SF fibers (Figure 3B) for the position of the Ser-C_α_ and Ser-C_β_ chemical shifts, which significantly moved up field, and for the presence of a carbonyl signal at 164.3 ppm, falling close to intense Gly C=O signal. The SF mat produced with ^13^C-enriched FA unequivocally confirmed the attribution of the new carbonyl signal to the C=O chemical shift of FA (Figure 3C) (Babij et al., 2016). Upon treatment with 3 M Na_2_CO_3_ (Figure 3D) the carbonyl signal significantly decreased in intensity but did not disappear completely, and the Ser-C_α_ and Ser-C_β_ signals shifted downfield, achieving a chemical shift like that of the reference SF fibers.

### 3.2 Chemical Characterization

The chemical structure of electrospun SF mats was preliminarily studied by determining the amino acid composition, with the aim to investigate whether the use of FA caused changes in the amino acidic pattern. As shown by the results listed in Table 1, the use of FA as solvent for obtaining SF nanofibers did not alter the amino acidic pattern of the protein, whose composition resembled that of native SF fibers used as reference material. Interestingly, also the SF mat treated with alkali overnight to remove FA (Kishimoto et al., 2018) did not show significant changes of the typical amino acidic pattern of SF. The amino acid composition of the SF mat and of the SF mat treated with 3 M Na_2_CO_3_ did not show a statistically significant difference with respect to the native SF fibers (*p* > 0.05).

**TABLE 1:** Amino acid composition (μmol/g ± S.D.).

Amino acid^a^	Native SF fibers	SF mat	SF mat treated with 3 M Na_2_CO_3_
Asp	216 ± 14	200 ± 5	213 ± 3
Thr	126 ± 2	115 ± 1	117 ± 1
Ser	1,402 ± 24	1,385 ± 1	1,376 ± 3
Glu	180 ± 20	185 ± 21	183 ± 1
Gly	6,189 ± 52	6,184 ± 14	6,247 ± 15
Ala	3,895 ± 7	3,909 ± 3	3,885 ± 3
Val	213 ± 28	190 ± 26	230 ± 6
Ile	83 ± 1	80 ± 3	64 ± 7
Leu	138 ± 1	129 ± 5	136 ± 9
Tyr	633 ± 19	650 ± 8	651 ± 1
Phe	94 ± 5	102 ± 6	84 ± 1
His	19 ± 3	32 ± 1	27 ± 1
Lys	52 ± 9	63 ± 1	54 ± 2
Arg	73 ± 3	69 ± 8	63 ± 1

a The amounts of each amino acid of the samples Native SF fibers, SF mat, and SF mat treated with 3 M Na_2_CO_3_ were not statistically different (*p* > 0.05).

As a second step of the chemical characterization, the FA content of SF mats was quantitatively determined by HS-GC/MS (Table 2). The total FA content was 247 ± 20 μmol/g. The overnight treatment with alkali caused a sharp decrease of FA, whose residual amount was less than 5% of the initial FA concentration, in good agreement with the spectroscopic results. The quantitative determination of FA was performed also on the SILKBridge^®^ nerve conduits and the results are reported in Table 2. The device showed a comparatively lower concentration of total FA with respect to the plain electrospun SF mats. This result is justified by the fact that only the electrospun component, whose weight accounts for about 60% of the device (Alessandrino et al., 2019), can be a potential source of leachable FA. In addition to the total FA, the so-called “free” FA was determined by analyzing the amount of solvent stripped from the sample under the conditions of incubation in the HS-GC vial, skipping the derivatization with sulfuric acid in 1-propanol. As shown in Table 2, the free FA is just a small fraction (about 3%) of the total FA contained in the device. Interestingly, the content of both total and free FA dosed on the two SILKBridge^®^ batches (A and B) did not show a statistically significant difference (*p* > 0.05).

**TABLE 2:** Quantitative determination of FA (μmol/g ± S.D.).

—	Total FA	Free FA
SF mat	247 ± 20	Nd^a^
SF mat treated with 3 M Na_2_CO_3_	11 ± 6	Nd
SILKBridge^®^ - Batch A^b^	166 ± 42	4.2 ± 1.5
SILKBridge^®^ - Batch B^b^	158 ± 52	5.3 ± 0.5

a Not determined.

b The amounts of total and free FA of Batch A and Batch B were not statistically different (*p* > 0.05).

### 3.3 Characterization of Alkali-Treated SF Mats

**FIGURE 4:**
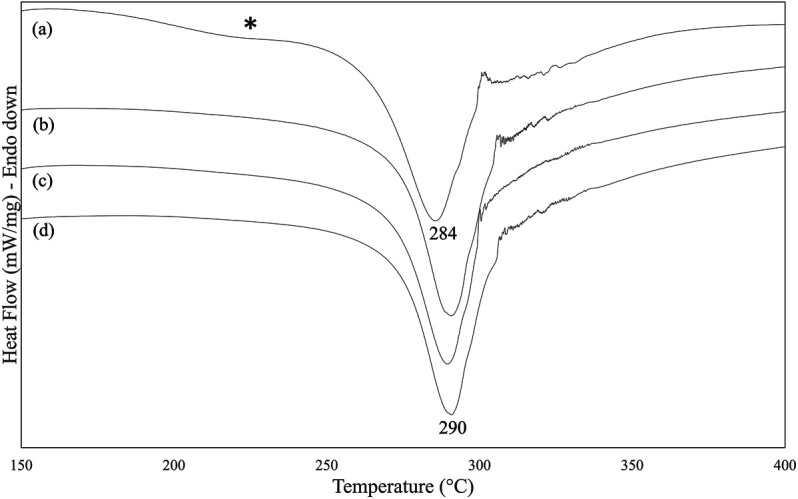
DSC thermograms of electrospun SF mat **(A)** and SF mats treated overnight with Na_2_CO_3_ at increasing concentration: **(B)** 0.75 M, **(C)** 1.5 M, **(D)** 3 M. The asterisk highlights the broad endotherm of thermally sensitive amorphous domains.

SF mats electrospun from FA-based dopes were prepared and characterized for their physical, mechanical, and biological properties before and after treatment with Na_2_CO_3_. The DSC analysis of SF mats highlighted the occurrence of some thermal changes upon treatment with alkali at increasing concentration, from 0.75 to 3 M Na_2_CO_3_ (Figure 4). The main melting/degradation peak shifted upwards from 284°C to 290°C and the broad low temperature endotherm centered at about 230°C almost completely disappeared. The former thermal event is attributed to the melting/degradation of regenerated SF with β-sheet crystalline structure (Marelli et al., 2010), while the latter reflects the thermally induced molecular motion of the SF chains above the glass transition temperature (Magoshi et al., 1977).

**FIGURE 5:**
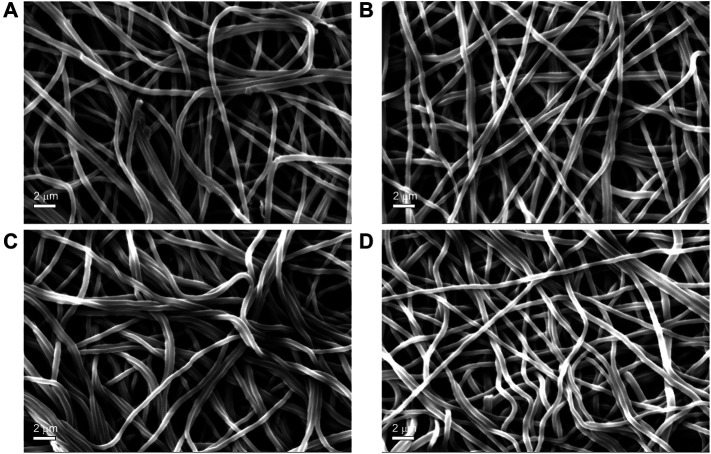
SEM morphology of electrospun SF mats untreated (Panel **A**) and treated overnight with Na_2_CO_3_ at increasing concentrations: 0.75 M (Panel **B**); 1.5 M (Panel **C**) **(D)** 3 M (Panel **D**) Magnification bar: 2 mm.

The results of the tensile measurements (Table 3) showed that the alkali-treated SF mats achieved a higher degree of stiffness, as demonstrated by the increase of the values of stress and modulus, and by the significant drop of strain. The results showed a statistically significant difference with respect to the untreated SF mat (*p* < 0.05). Also, the morphology of SF mats was affected by the alkali treatment. While the overall fibrous structure was well preserved (Figure 5), the average fiber diameter suddenly increased upon alkali treatment with statistical significance (*p* < 0.05), without a clear dependence on alkali concentration (Table 4). This trend was macroscopically reflected by a tendency of the alkali-treated mats to shrink after washing and drying.

**TABLE 3:** Tensile properties of electrospun SF mats.

	SF mat	SF mat treated with 3 M Na_2_CO_3_	*p* Value
Stress (MPa)	2.55 ± 0.21	3.37 ± 0.14	<0.05^a^
Strain (mm/mm)	1.04 ± 0.09	0.78 ± 0.08	<0.05
Modulus (Mpa)	5.46 ± 0.62	7.03 ± 0.75	<0.05

a The values of stress, strain, and modulus were statistically different.

**TABLE 4:** Average diameter of electrospun SF fibers.

	SF mat	SF mat treated with Na_2_CO_3_ ^a^		
		0.75 M	1.5 M	3 M
Diameter (nm) ± S.D.	440 ± 72	484 ± 78	476 ± 72	492 ± 85
Number of fibers (n)	173	195	188	196

a The values of fiber diameter of the samples treated with 0.75, 1.5, and 3 M Na_2_CO_3_ were statistically different from the reference untreated SF mat (*p* < 0.05).

**FIGURE 6:**
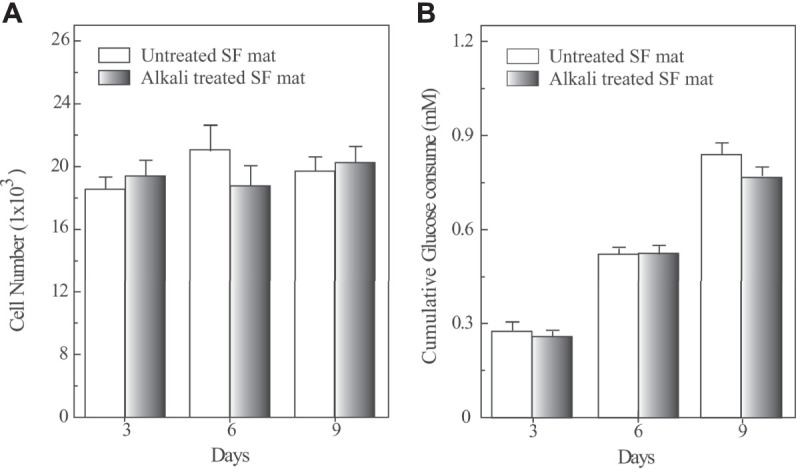
Quantification of cell numbers (panel A) and cumulative glucose consumption (panel B) of HADFs seeded onto untreated electrospun SF mat or onto alkali-treated electrospun SF mat as a function of culture time. Panel **A**: Cell numbers were determined by the Quant-iT PicoGreen dsDNA Kit (see Materials and Methods for details). A standard curve based on known concentrations of DNA was used to assess the cell numbers. Panel **B**: HADFs adhering on both kinds of electrospun SF mats exhibited an alike metabolic rate of glucose consumption. The results were normalized per 1,000 cells. Data are the mean values of three distinct experiments, each in triplicate. No significant statistical differences occurred (*p* > 0.05).

The biocompatibility of SF mats was studied *in vitro* with HADFs cells (Figure 6). During the 9 days of culture HADFs seeded onto untreated and alkali-treated SF mats did not significantly increase their numbers. The mean cellular densities at day 9 were 313 ± 18 and 309 ± 23 cells/mm^2^ of apparent surface area for untreated and alkali-treated SF mats, respectively. These figures did not significantly differ from each other (*p* > 0.05). The results concerning the glucose consumption as an energy source normalized per 1,000 cells showed that the cumulative consumptions of glucose on either kind of SF mats steadily increased and did not significantly differ from each other at different times (*p* > 0.05).

## 4 Discussion

### 4.1 Spectroscopic Evidence of the FA-SF Bonding in Electrospun Mats

FA is highly volatile (vapor pressure: 42.71 hPa at 20°C) and the greatest part of it is eliminated during electrospinning before the fibers reach the collector. However, residual FA is incorporated into the electrospun mat and remains there even after the consolidation/washing steps usually performed with hydroalcoholic solutions, as documented by the FTIR (Figure 1), Raman (Figure 2), and ^13^C CP/MAS NMR spectra (Figure 3). All spectroscopic analyses showed distinct spectral changes attributable to presence of FA in the SF mat. The shift of the Raman carbonyl band from 1,707 cm^−1^ in pure FA (Figure 2D) up to 1,725 cm^−1^ in the SF mats (Figure 2A) suggests the occurrence of a strong chemical interaction between FA and SF. The strength of the interaction was qualitatively confirmed by its resistance to dissolution/dialysis/casting treatments (Figure 1C), as well as by the fact that, even upon overnight alkali treatment, the intensity of the carbonyl band decreased significantly but never disappeared completely (Figure 1B, Figure 2B, and Figure 3D).

The appearance of the new carbonyl stretching bands in FTIR and Raman and of the new carbonyl signal in NMR was accompanied by changes in other spectral regions, i.e.: 1,200-1,000 cm^−1^ for FTIR and Raman, and 50–60 ppm for NMR. These changes provide important information about the type of the interaction between FA and SF. The up-field shift of the Ser-C_α_ NMR signal, the weakening of the FTIR band at 1,064 cm^−1^ (νC-O of Ser), and the strengthening of the IR band at 1,168 cm^−1^ (1,171 cm^−1^ in Raman) strongly supports the occurrence of an esterification reaction between FA and the OH side groups of Ser and Thr residues (Kienhuis et al., 1959; Narita, 1959; Smillie and Neurath, 1959; Dollish et al., 1973). In fact, the band at 1,168 cm^−1^ has been assigned to the asymmetric νC-O-C of formyl esters of Ser/Thr in proteins, like lysozyme (Kienhuis et al., 1959). Also, the position of the νC=O band at 1,725 cm^−1^ is consistent with the formation of formyl esters (Dollish et al., 1973). Upon alkali treatment, all spectroscopic data agreed in highlighting that the Ser/Thr associated bands regained the typical position and intensity of SF materials that have never been in contact with FA, indicating that the formyl ester was hydrolyzed, and FA was removed, although not exhaustively.

### 4.2 Chemical and Structural Implications of the FA-SF Bonding in Electrospun Mats

The spectroscopic data consistently showed that the OH groups of Ser and Thr underwent esterification with FA. The amino acid analysis of the SF mat reported in Table 1 did not allow to calculate the yield of esterification because, as expected, the ester bond formed by FA with the OH groups of Ser and Thr was hydrolyzed under the strong acidic conditions used for the preparation of the hydrolysate, so that both Ser and Thr regained their free state and were dosed as free amino acids. Nevertheless, if the theoretical amount of hydroxy amino acids available for binding FA (Ser + Thr ≅ 1,500 μmol/g; Table 1) is compared to the total amount of FA dosed by HS-GC/MS (247 μmol/g; Table 2), it can be deduced that only a part of the available OH groups (about 17%) was involved in the formation of formyl esters. This evaluation, based on the chemical analysis, agrees with another one obtained from the IR spectra through the calculation of the A_1725_/A_1447_ intensity ratio which gave an amount of reactive OH groups of about 19% (Supplementary Material) (Kienhuis et al., 1959). Considering that the amount of FA is not a limiting factor in the electrospinning dope, this relatively low esterification yield may account for inherent steric constraints which restricted the reaction of FA with the OH groups potentially available along the SF chains.

A careful inspection of the spectroscopic data of SF mats before and after alkali treatment can help to clarify this aspect. The FTIR, Raman, and NMR spectra are all characteristic of an SF material with β-sheet crystalline structure (Magoshi et al., 1977; Saito et al., 1984; Tsukada et al., 1994; Edwards and Farwell, 1995; Monti et al., 1998; Monti et al., 2001). The β-sheet crystals were little, if at all, affected by the alkali treatment. The FTIR crystallinity index (CI), calculated from the intensity ratio between the two Amide III components at 1,230 cm^−1^ and 1,260 cm^−1^ (Marelli et al., 2010), gave a CI value of 0.59 ± 0.03 and 0.60 ± 0.02 before and after alkali treatment, respectively. The relative intensity of the IR bands at about 1,000 cm^−1^ and 976 cm^−1^, assigned to Ala-Gly sequences comprising the low complexity “crystalline” blocks did not change (Frushour and Koenig, 1975). The peak position of the strong Amide I and Amide II bands remained constant at 1,627 cm^−1^ and 1,515 cm^−1^, respectively. Only the high wavenumber components of Amide I at about 1,690-1,660 cm^−1^, assigned to vibrations of unordered and β-turns domains of SF, slightly decreased in intensity, suggesting an increase of molecular order of the material (Hu et al., 2006). The latter observation is also supported by the Raman data. The full width at half maximum of Amide I slightly decreased, suggesting a change towards a more conformationally homogeneous structure. Accordingly, the β-sheet sensitive bands at 1,162, 1,085, 978, and 884 cm^−1^ suggested an overall slight increase of molecular order.

All these spectral details allow concluding that the local molecular rearrangements occurring upon FA removal by alkali preferentially involved the amorphous SF domains, which achieved a more ordered conformational structure, while the β-sheet crystals were almost unaffected (Pavoni et al., 2016; Taddei et al., 2017). This observation leads us to suggest that during the process of fiber formation by electrospinning the solvent was squeezed away from the regions where the SF chains established strong intermolecular interactions upon coagulation with formation of stable β-sheet crystals, while only the fraction of FA remaining in the less ordered domains could react with the available OH groups of Ser and Thr forming formyl esters. This mechanism may account for the relatively low yield of esterification calculated from the total amount of theoretically available Ser/Thr residues.

If the primary structure of the Heavy and Light Chains of SF is considered, it emerges that Ser is the third most abundant amino acid, accounting for a total number of 661 residues, of which 636 are regularly distributed along the sequence of the Heavy Chain (UniProtKB - P05790) (Zhou et al., 2000; Zhou et al., 2001) and 25 are in the Light Chain (UniProtKB - P21828) (Yamaguchi et al., 1989). Thr is much less abundant than Ser, accounting for 66 residues considering both the Heavy and Light SF Chains. About 75% of the total number of Ser/Thr residues are located in the 12 low-complexity, highly repetitive blocks, which are mainly involved in the formation of the β-sheet crystalline domains, while the remaining residues appear in the 11 amorphous blocks, as well as in the N- and C-terminal segments (Yamaguchi et al., 1989; Zhou et al., 2001). Thus, the total number of Ser/Thr residues lacking significant steric constraints for the esterification to occur decreases from 1,500 μmol/g to about 370–380 μmol/g, which raises the yield of reaction up to about 65%.

### 4.3 Is the Removal of FA From the Electrospun SF Mats a Viable Option?

The treatment of SF mats with aqueous Na_2_CO_3_ is effective in removing SF-bound FA (Kishimoto et al., 2018). The execution of this treatment significantly reduced the amount of FA in the SF mat (Table 2), without any apparent negative consequence on the chemical structure of the material (Table 1). Moreover, the cumulative spectroscopic results demonstrated that the crystalline structure of SF mats was not affected by alkali treatment. The major conformationally sensitive bands displayed only minor changes, all accounting for chain rearrangements occurring in the less ordered SF domains.

Although SF is reported to be resistant to alkali more than other protein fibers, owing to its peculiar chemical structure (Scheibel et al., 2016), it is not trivial to verify whether the effect of an alkali treatment can alter the intrinsic properties of electrospun SF mats. To answer this issue, the mats were further characterized by means of morphological, thermal, and mechanical analyses. The immersion in alkali did not alter the morphology of the electrospun mat, whose fibrous texture was well preserved (Figure 5). The most significant change was the increase of the average diameter of electrospun fibers (Table 4). Upon immersion in alkali, the fibers underwent swelling, a phenomenon that increased their volume. Fiber swelling is typically anisotropic, with transverse swelling largely exceeding in intensity the longitudinal one (Morton and Hearle, 2008). Swelling is reversible and is usually recovered on drying, although a net fiber contraction may occur. This effect has been demonstrated for wet spun regenerated silk fibers (Plaza et al., 2008), and the same likely occurred for the electrospun fibers of alkali-treated SF mats upon drying. Therefore, at the microscopic level the average fiber diameter increased, while macroscopically a strong tendency of the mat to shrink was observed. The increase of the values of stress and modulus and the drop of strain accounted for these morphological and structural changes and for the higher level of mechanical stiffness achieved by the alkali-treated mats (Table 3). The thermal changes observed by DSC analysis (Figure 4) indicated an overall restriction of the molecular motion of the SF chains. In summary, all these morphological, physical, and mechanical changes are in keeping with the spectroscopic evidence of a more compact molecular organization achieved by the amorphous domains of SF mats upon alkali treatment. It is difficult to predict only based on these results if these changes could be favorable or unfavorable in terms of the property/performance balance of the electrospun SF mat. Any conclusion about this issue strictly depends on the intended biomedical application and could only be verified through field testing by means of suitable *in vivo* assays. In general, it can be outlined that even if these changes were deemed low impact, the execution of such an alkali treatment would burden the production process of the scaffold both from the point of view of processing time and costs.

If we consider only the biocompatibility data (Figure 6), it is possible to draw the conclusion that the removal of FA from SF mats doesn’t seem a mandatory option. In fact, cell adhesion and proliferation were not adversely affected by the presence of SF-bound FA, in good agreement with the results obtained from widely different *in vitro* models like human keratinocytes and fibroblasts (Min et al., 2004), L929 murine fibroblasts (Marelli et al., 2009), mouse osteoblast-like cells MC3T3-E1 (Meechaisue et al., 2007; Ki et al., 2008; Park et al., 2010), NIH 3T3 fibroblast (Ki et al., 2007; Marelli et al., 2010; Kishimoto et al., 2018), and murine bone marrow derived mesenchymal stem cells (Griffanti et al., 2019). Also a number of *in vivo* studies, where FA-based SF nanofibrous scaffolds were implanted in different niches, such as subcutaneously in Lewis rats (Catto et al., 2015), as a small caliber vascular graft into the abdominal aorta of Lewis rats (Cattaneo et al., 2013), as patches in the treatment of diabetic wounds of db/db diabetic mice (Navone et al., 2014), as nerve guide for the repair of a 5 mm facial nerve defect in Sprague-Dawley rats (Hu et al., 2013), as scaffold for bone regeneration (Kim et al., 2005; Park et al., 2010), did not raise biocompatibility concerns.

However, this conclusion may appear quite trivial if viewed in the context of a translational process aimed at bringing a medical device from lab to clinic. The correct answer could only come from the implementation of a structured biological evaluation program within a risk management process in accordance with mandatory regulations addressing the biological safety of medical devices intended for use in humans. In particular, the ISO 10993-17 standard provides guidance through a step-by-step process aimed at evaluating the risks associated with exposure to hazardous leachable substances released by a medical device at the site of implantation.

### 4.4 Evaluation of the Toxicity of FA Released From Electrospun SF Mats

In this paragraph, the toxicological evaluation of the impact of residual FA left by the electrospinning process is presented within a clinical setting perspective, using the SILKBridge^®^ nerve conduit as case study. This medical device is currently under evaluation in a first-in-human clinical trial (ClinicalTrials.gov identifier: NCT03673449) to assess its safety and performance for the reconstruction of digital nerves in humans. The wall of the conduit has a hybrid structure comprising two electrospun layers which encase a textile layer made of native SF fibers (Alessandrino et al., 2019). Only the electrospun component, whose weight accounts for about 60% of the device, can be a potential source of leachable FA. As discussed above, the greatest part of FA is bound to Ser/Thr residues as formyl ester, while only a small fraction (about 3%) is present as free FA (Table 2).

In terms of hazard classification (Regulation EC No 1272/2008), FA is a recognized harmful substance because of its acidic/corrosive properties. It causes severe skin burns and eye damage, it is toxic if inhaled and corrosive to the respiratory tract. For these reasons, FA is listed as medium-to-high hazard for workers coming in contact with this compound. The general population may meet FA in consumer products. In fact, FA can be used as fragrance ingredient, preservative, and pH adjuster in cosmetic products for hair care, as a component of synthetic flavoring substances and adjuvants added to food for human consumption, or active ingredient in over-the-counter drug products. Johnson et al. (2016) recently reviewed the toxicity profile of FA. Toxicological data about FA are also available in a number of databases, including the European ECHA database (European Chemical Agency, https://echa.europa.eu/home). Reviewing the different entries that make up the toxicological profile of FA it is possible to deduce that it is not considered to be a skin sensitization substance. No genotoxicity was observed in all *in vitro* and *in vivo* tests. Accordingly, all the tests aiming at verifying carcinogenicity, reproductive and developmental toxicity gave negative results.

Owing to its chemical properties (pK_a_ = 3.7 at 20°C), FA rapidly dissociates to formate anion in aqueous solutions at near neutral pH or in body fluids at physiological pH values. The toxicokinetic behavior, metabolism, and elimination *in vivo* of the formate anion has been investigated in several species including humans (Johnson et al., 2016). Formate does not persist or accumulate in the tissues, but it undergoes metabolic oxidation to carbon dioxide in the liver and erythrocytes, primarily *via* the folate-dependent pathway. The toxicological profile of formate is similar to that of FA but lacks the local acute toxicity implications associated to the acidity and corrosive power of the acid. An exogenous source of formate is the ingestion/inhalation of methanol, which is metabolized *via* formaldehyde, further oxidized to formate and then eliminated. Interestingly, formate can also be of endogenous origin, playing a role in the cellular and whole-body metabolism of mammals (Brosnan and Brosnan, 2016). Mammalian cells produce formate from the oxidation of the third carbon of Serine using either a cytosolic or mitochondrial pathway (Oizel et al., 2020; Pietzke et al., 2020). Both pathways can sustain the one-carbon demands of cell proliferation for the synthesis of nucleotides and methyl groups, but the mitochondrial pathway is essential for formate overflow, whose increase has been proposed as potential biomarker for certain cancers of oxidative nature (Meiser et al., 2018).

In a toxicological risk assessment perspective, the largest amount of a leachable substance that is deemed acceptable on a daily basis through exposure to a medical device must be determined. The results of the chemical analysis of the SILKBridge^®^ nerve conduit (Table 2) represent the starting point to perform the toxicological evaluation following the provisions of ISO 10993-17. As a first step, the concentration of FA expressed in μmol/g was transformed in μg/device to obtain the total amount of FA released by one device (Table 5). Then, assuming a worst-case scenario of multiple devices implanted at the same time in one patient (one per finger, i.e. 10 devices), the value was multiplied by 10 to calculate the Potential Patient Daily Intake (PPDI, expressed in mg/day). The PPDI value gives the total amount of chemical to which the patient is potentially exposed during the lifetime. A further worst case assumption in the toxicological risk assessment is that the total amount expressed by the PDDI value is released just in 1 day. The toxicological data about FA retrieved from the database allowed setting the value of Tolerable Exposure (TE, expressed in mg/day; Supplementary Material), i.e. the amount of chemical agent that doesn’t pose concerns for human health. Finally, the TE/PPDI ratio was calculated to obtain the Margin of Safety (MoS). For values of MoS ≤1 the compound is considered to pose a toxicological concern. For values of MoS >1 the compound does not raise risks for human health. We adopted a more conservative approach setting a threshold for MoS ≥10. As can be observed in Table 5, despite the worst-case assumptions made at different levels of the toxicological evaluation, i.e. total amount of FA released in 1 day, multiple devices (up to 10), MoS threshold higher (≥10) than recommended by the reference standard (>1), FA cannot be held responsible for causing toxicological concerns following implantation of the SILKBridge^®^ nerve conduit in humans.

**TABLE 5:** Toxicological analysis of leachable FA according to ISO 10993-17.

FA concentration (μmol/g)	Total released amount (μg/device)	Potential patient daily intake (PPDI) (mg/day)	Tolerable exposure (TE) (mg/day)	Margin of safety (MoS)
209^a^	240^b^	2.4^c^	35.4^d^	15^e^

a As worst-case scenario, the highest values of FA concentration of the two batches, returned by the “mean +S.D.” value (Table 2), were averaged and used for the toxicological analysis.

b An average weight of 25 mg was considered for the SILKBridge^®^ Device. MW, of formic acid: 46.03.

c Worst-case scenario of 10 devices implanted at the same time in one patient.

d The calculation of TE, is detailed in Supplementary Material.

e MoS = TE/PPDI.

Regarding the rate of FA release into the surrounding tissues, a likely scenario is that, upon swelling in the first period after implantation, the free FA fraction gradually diffuses out of the electrospun component of the device wall. The greatest part of FA, which is retained in the device in form of Thr/Ser formyl ester, could be released at later stages, following the progressive degradation of the electrospun layers exposed to the hydrolytic/oxidative environment created by the inflammatory response at the site of implantation (Biggi et al., 2020; Fregnan et al., 2020). In any case, it must be considered that the release will never occur in a bursting way, but gradually over time and in a more physiologically compliant manner, so that the local load is diluted over time. This assumption is supported by the fact that SILKBridge^®^ is made of silk fibroin materials characterized by a slow rate of degradation, i.e. from months to years for regenerated electrospun fibers and native microfibers, respectively (Biggi et al., 2020; Fregnan et al., 2020).

In conclusion, the results of the toxicological evaluation demonstrated that the amount of residual FA coming from the manufacturing process of the SILKBridge^®^ nerve conduit is under control and that it is largely below the threshold for toxicological concern, despite having considered multiple worst-case scenarios, from the possibility of multiple implants, to the ten-fold increase of the acceptable value of Margin of Safety, and the assumption that the total amount is released in 1 day in a bursting way.

## 5 Conclusion

The combined use of different spectroscopic techniques (FTIR, Raman, ^13^C CP/MAS NMR) allowed investigating in detail the nature of the interaction between SF and FA in electrospun mats. In the presence of FA, a fraction of the hydroxyl groups of Ser and Thr residues (about 17% of the 1,500 μmol/g of hydroxy amino contained in SF) underwent esterification resulting in the formation of formyl esters. Considering the primary structure of the SF Heavy and Light Chains, as well as the conformational details highlighted by the spectroscopic results, it was possible to conclude that the esterification reaction preferentially involved the amorphous SF domains.

The HS-GC/MS analyses allowed determining the total amount of FA contained in the SF mat (247 ± 20 μmol/g). The greatest part was present as formyl ester, while a very small part (about 3%) was present as free acid. The treatment of SF mats with an alkaline solution of Na_2_CO_3_ removed most of the bound FA. However, the removal of FA by alkali treatment raised some concerns about the impact that the morphological, physical, and mechanical changes might have on the performance of the material, thus questioning the real need to perform this type of post-treatment on a routine basis for removing FA from SF mats. In fact, upon alkali treatment, the average diameter of the electrospun fibers increased from about 440 nm to about 480 nm, the mat shrunk, became stiffer (the modulus increased from about 5.5 MPa to about 7 MPa), and lost elasticity (the strain decreased from about 1 mm/mm to about 0.8 mm/mm). The biocompatibility of the SF mat, evaluated on the basis of proliferation of human adult dermal fibroblasts (313 ± 18 and 309 ± 23 cells/mm^2^ for untreated and alkali-treated SF mat, respectively) and metabolic activity did not change significantly.

Finally, only the adoption of a rigorous approach to the toxicological analysis of leachable substances, as imposed by the stringent regulations in place for the assessment of biological risk related to the use of implantable medical devices, allowed to settle the issue of health risks associated with the presence of FA. Using the SILKBridge^®^ nerve conduit as case study and following the provisions of the ISO 10993-1 standard, the Potential Patient Daily Intake of 2.4 mg/day was calculated from the total amount of FA determined by HS-GC/MS. The toxicological data about FA retrieved from the toxicological databases allowed setting the value of Tolerable Exposure to 35.4 mg/day. A value of the Margin of Safety of 15 was obtained, which largely exceeded the threshold of MoS = 1 above which the compound does not raise risks for human health. This leads to the conclusion that the FA still present within the electrospun component of the device wall and possibly released into the surrounding tissues after implantation cannot raise toxicological concerns for human health.

## Data Availability

The original contributions presented in the study are included in the article/Supplementary Material, further inquiries can be directed to the corresponding author.
