# Supplementary material for: Electrospun Silk Fibroin Scaffolds for Tissue Regeneration: Chemical, Structural, and Toxicological Implications of the Formic Acid-Silk Fibroin Interaction
Source: Front Bioeng Biotechnol. 2022 Jan 27;10:833157. doi: 10.3389/fbioe.2022.833157 (PMC8829063; doi:10.3389/fbioe.2022.833157)
Supplement: Supplementary file 1 [file DataSheet1.docx]

Supplementary Material

# Calculation of the FTIR A_1725_/A_1447_ intensity ratio

A_1725_/A_1447_ intensity ratios were calculated as peak heights from the IR spectra reported by Kienhuis et al. (1959) for lysozyme treated with FA and from the IR spectra reported in this study for the SF mat. For the lysozyme treated with FA the authors reported a percentage of reacted Ser and Thr residues of 100%. This information has been utilized to calculate the percentage of reacted Ser and Thr residues in the SF mat from the IR A_1725_/A_1447_ intensity ratio. Interestingly, this percentage showed a good agreement with the HS-GC/MS data (19% *versus* 17%).

Table S1.

| Sample | A_1725_/A_1447_ ratio | % of reacted Ser and Thr residues |
| --- | --- | --- |
| Lysozyme treated with FA ^(*)^ | 2.81 | 100% |
| Untreated SF mat,  this study | 0.53 | 19% |

^(*)^Kienhuis, H., Blasse, G., Matze, J. (1959). Action of anhydrous formic acid on peptides and proteins. Nature 184:2015-2016. Doi: 10.1038/1842015a0

# Toxicological risk assessment of formic acid (FA)

The toxicological evaluation of FA was carried out to evaluate the potential toxicological concerns related to the release of the compound from the medical device SilkBridge^®^. The final evaluation was achieved by combining the results of the chemical analysis by HS-GC/MS with the toxicological data from literature, the clinical use of the device, and considering adults as target population.

The toxicity assessment reviewed the scientific information concerning the toxicological properties of FA. Both systemic and local effects were addresses in this part of the study.

The systemic toxicity endpoints covered in the risk assessment review were as follows:

- genotoxicity
- acute, sub-acute, sub-chronic and chronic systemic toxicity
- toxicity to reproduction and developmental toxicity
- carcinogenicity
- toxicokinetics
- biodegradation
- neurotoxicity
- immunotoxicity

The toxicological profile was then completed by considering also the following local toxicity endpoints:

- skin irritation and skin sensitization

Toxicological data for FA were searched using databases such as ToxPlanet, PubChem, PubMed, OECD Existing Chemicals Database, COSMOS Space, ECHA - European Chemical Agency, FDA - Food and Drug Administration, etc.) and are summarized in Table S2.

Table S2. Summary of the toxicological information on FA.

| Endpoint | Results |
| --- | --- |
| Skin irritation and corrosion | Corrosive |
| Skin sensitization | Negative |
| Genotoxicity | Negative |
| Toxicity to reproduction | Negative |
| Carcinogenicity | Negative |

On the basis of the toxicological data collected for each endpoint, the approach described in ISO 10993-17:2002 was applied to derive the value of “No Observed Adverse Effect Level” (NOAEL, expressed in mg / kg of body weight / day), which is the greatest amount of a substance found by experiment or observation which causes no detectable adverse alteration of morphology, functional capacity, growth, development or life span of the target organism under defined conditions of exposure.

Then, the Tolerable Intake (TI) value for non-cancer endpoints, as is the case of FA, was calculated from the following formula:

TI = NOAEL / (UF_1_ • UF_2_ • UF_3_)

Where UF_1_, UF_2_, and UF_3_ are Uncertainty Factors used to correct the NOAEL value for different variability sources. In particular, UFs are defined as follows:

- UF_1_: inter-individual variability in the human population (range: 1-10; default value: 10)
- UF_2_: inter-species extrapolation (range: 1-10; default value: 10)
- UF_3_: quality and relevance of the experimental data (range 1-100; set value: 10)

From TI the Tolerable Exposure (TE) value was dericìved according to the formula:

TE = TI • BW

Where: BW is the body weight, set per default as 50 kg for adult population according to EMA/CHMP/CVMP/ SWP/169430/2012. TE is expressed in mg / day.

Table S3 shows the step-by-step path to calculate the value of TE.

Table S3. Tolerable Exposure calculation

| NOAEL  [mg/kg BW/day] | UF_1_ | UF_2_ | UF_3_ ^(1)^ | TI  [mg/kg BW/day] | BW  [kg] | TE  [mg/day] |
| --- | --- | --- | --- | --- | --- | --- |
| 708 | 10 | 10 | 10 | 0.708 | 50 | 35.4 |

^(1)^ The uncertainty factor UF_3_ of 10 was applied given the differences in clinical use of the device and the characteristics of the selected study (oral administration in rats; conservative approach).
